# A new parafocusing paradigm for X-ray diffraction

**DOI:** 10.1107/S1600576720008651

**Published:** 2020-07-24

**Authors:** Danae Prokopiou, James McGovern, Gareth Davies, Simon Godber, Paul Evans, Anthony Dicken, Keith Rogers

**Affiliations:** a Cranfield Forensic Institute, Cranfield University, Shrivenham SN6 8LA, UK; b HALO X-ray Technologies, Nottingham Trent University, Nottingham NG11 8NS, UK; c The Imaging Science Group, Nottingham Trent University, Nottingham NG11 8NS, UK

**Keywords:** focal construct geometry, powder X-ray diffraction, Bragg–Brentano geometry, conical incident beams

## Abstract

A new approach to parafocusing X-ray diffraction implemented with an annular incident beam is demonstrated for the first time. The method exploits an elliptical specimen path on a flat sample to produce relatively high intensity maxima that can be measured with a point detector. It is shown that the flat-specimen approximation tolerated by conventional Bragg–Brentano geometries is not required. A theoretical framework, simulations and experimental results for both angular- and energy-dispersive measurement modes are presented and the scattering signatures compared with data obtained with a conventional pencil-beam arrangement.

## Abstract 2

A novel approach to parafocusing X-ray diffraction with an annular incident beam is demonstrated. Proof of principle is achieved via a theoretical approach, simulations and experiments, demonstrating significantly higher intensity scattering when compared with conventional Bragg–Brentano geometries, but without the need for flat-specimen approximations.

## 1. Introduction   

In general, instruments developed for acquisition of diffraction data from polycrystalline specimens are diffractometers that exploit Bragg–Brentano parafocusing geometry. The term parafocusing is in common use in this context to describe the non-ideal focusing arising from a finite source width, specimen transparency *etc*. (Jenkins & Snyder, 1996 ▸). To some extent, the acquisition of diffraction data has found application areas beyond the laboratory, although modifications to fundamental instrumentation are rare. For applications such as materials process control, speed of data acquisition whilst maintaining signal fidelity (*e.g.* high signal-to-noise ratio) is critical and, although more sensitive detectors and brighter X-ray sources go some way to addressing these issues, costs may be prohibitive. An alternative approach may be to modify the X-ray beam topology, and such a method is explored below.

For conventional data collections, a divergent incident beam passes from the X-ray tube through a divergence slit or pinhole and illuminates a flat surface specimen with an incident angle θ (Aslanov *et al.*, 1998 ▸; He, 2009 ▸). Coherently scattered X-rays leave the surface of the specimen at an angle 2θ from the incident beam, pass through an antiscatter slit and converge on a receiving slit (He, 2009 ▸). Parafocusing arises when the radial distance between the X-ray source and the specimen is equal to that between the specimen and the receiving slit. To achieve parafocusing, the specimen surface should be curved to coincide with the radius of curvature of the focusing circle. A flat specimen causes defocusing (peak broadening), which occurs especially at high values of θ, although this is mitigated by minimizing the beam footprint along the specimen. In a θ:2θ arrangement, the specimen is rotated by θ whilst the detector is rotated by 2θ during data collection. Data can also be collected with a θ:θ goniometer, where the specimen is stationary in the horizontal position and both the X-ray tube and the detector move simultaneously over the angular range θ. Extending the beam footprint (for greater specimen volume averaging) whilst maintaining parafocusing can be achieved by extending the beam length normal to the measurement plane (along the rotation axis), but this incurs several penalties including the requirement for a larger-area detector and other optical elements such as Soller slits. In contrast, our new approach maintains the parafocusing even for flat specimens, significantly increases the beam footprint and enables intensity measurement with a point detector.

As an alternative to a pencil or line-shaped incident beam, we propose a topological modification of the incident beam into a right elliptical, hollow cone. This topology was previously suggested for use in transmission diffraction intensity acquisition (Rogers *et al.*, 2010 ▸). Referred to as focal construct geometry (FCG), this was shown to cause Debye cones to converge at single locations along the incident cone’s primary axis (Evans *et al.*, 2014 ▸), producing ‘condensation’ points of relatively high intensity (Evans *et al.*, 2010 ▸; Rogers *et al.*, 2012 ▸; Prokopiou, 2014 ▸; Prokopiou *et al.*, 2017 ▸; Dicken *et al.*, 2018 ▸). We submit that a similar intensity advantage may also be produced by adapting FCG principles to support reflection mode operation. To achieve this goal, it will be critically important to maintain true parafocusing from flat specimens over significantly longer specimen paths.

## 2. Theoretical development   

**Figure 1:**
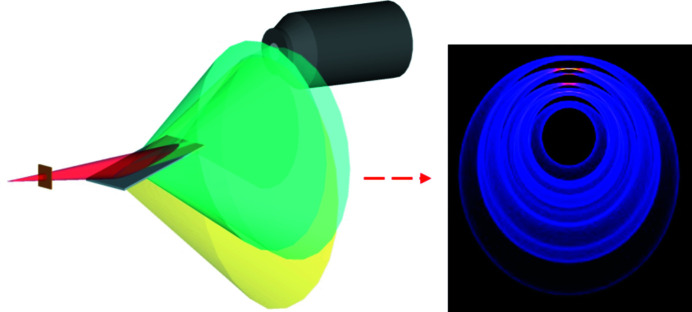
3D schematic of a parafocusing reflection FCG arrangement, illustrating the convergence of four scattering maxima arising from the extreme points along the incident beam’s footprint onto the specimen, and a simulated example of the scattering distribution from a 0.5 mm-thick corundum specimen.

In contrast to transmission FCG experiments, reflection FCG (shown in Fig. 1 ▸) produces diffraction maxima in similar spatial positions to conventional Bragg–Brentano geometry. The incident beam may be considered as an oblique hollow cone forming an elliptical beam footprint on the specimen surface and with positions of the diffraction maxima dependent upon experimental features such as the incident beam opening angle, specimen rotation (degree, direction and rotation axis), specimen position and material characteristics (*i.e.* lattice spacings).

**Figure 2:**
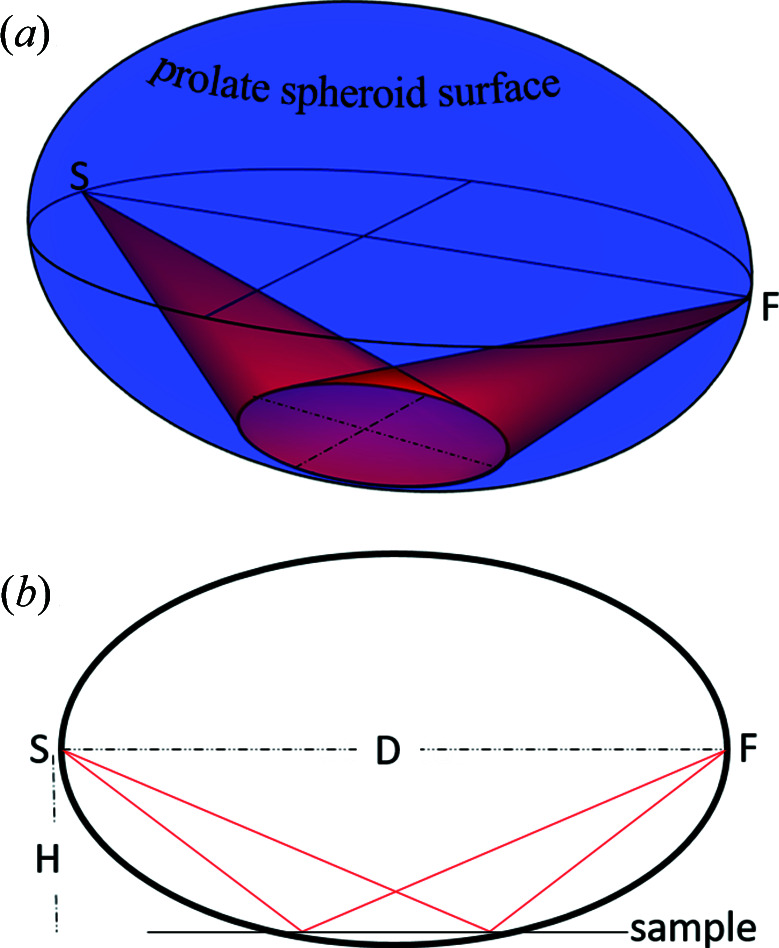
(*a*) The surface that satisfies conditions for parafocusing at a specific scattering angle where the X-ray source, S, detector position, F, and elliptical footprint on a planar specimen are indicated. (*b*) A cross section through the prolate spheroid for clarity. Note this is not a focusing circle.

The parafocusing associated with this novel geometry may be derived as follows. Consider the usual parafocusing geometry for a single scattering angle (2θ) where the source and detector are placed upon a focusing circle and chords define incident and diffracted beams (see Fig. 2 ▸). The 3D surface that then defines all points satisfying the parafocusing condition is a prolate spheroid. The intersection of this spheroid with any planar specimen surface defines an X-ray beam footprint such that every point has the same scattering angle, 2θ. For a planar specimen surface, this footprint is therefore an ellipse and this enables ideal parafocusing (no flat-specimen approximation) from any such specimen.

The minor and major axes of any elliptical footprint can be calculated simply from geometrical considerations following two assumptions: (*a*) the minor axis is located midway between source and detector and (*b*) the major axis is parallel to the source–detector vector. In this case, which is typical of reflection parafocusing geometries, the major axis of the beam footprint, *a*, can be calculated from
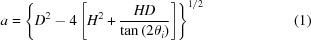
and the minor axis, *b*, from
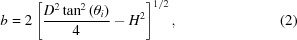
where *D* is the source–detector distance, *H* is the perpendicular height of the source and detector from the specimen plane, and 2θ_*i*_ is the angle of scatter for the *i*th reflection.

Therefore, ideal parafocusing can occur for an elliptically extended beam incident upon a flat specimen. Furthermore, satisfying the above conditions for any Bragg reflection will produce a single point of intersection for all Debye cones of that reflection originating from the incident beam footprint.

Forming an elliptical footprint of appropriate dimensions on the specimen surface can be achieved through the imposition of suitable collimation such as a plate absorber with an elliptical annular X-ray-transparent region. Such a collimator placed parallel to the specimen surface, and at a distance *H*_c_ from the specimen, would have major, *a*_c_, and minor, *b*_c_, axes defined as
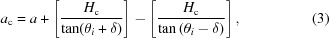


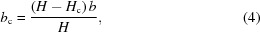
where 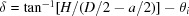.

An important implication of the above analysis is that the footprint and therefore collimator eccentricities are functions of scattering angle. We have explored how this difficulty may be overcome, whilst retaining parafocusing, through modifications to both angular- and energy-dispersive approaches. These are discussed in detail below.

**Figure 3:**
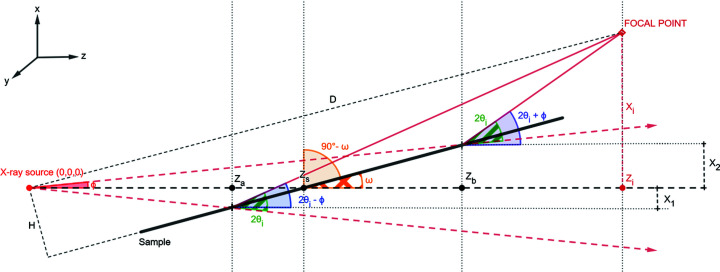
Geometric schematic for the calculation of 2θ_*i*_ dependent upon the detector position (*X_i_*, *Z_i_*) along the focusing arc when the specimen is fixed at an angle ω.

To apply the new topology to angular-dispersive measurement, a circular annular collimator may be employed and the specimen tilted to a fixed position producing an annular elliptical interaction volume such that the major and minor axes are consistent with the requirements of equations (1) and (2). The parafocusing condition is then maintained for a continuum of scattering angles in a single plane (*e.g.* *xz* if the specimen rotation is around the *y* axis, as shown in Fig. 3 ▸). For example, in the case of a specimen tilt around the *y* axis, a detector would be translated according to the coordinates
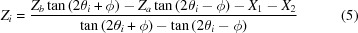
and
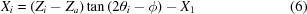
when 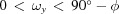 and 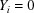.

If 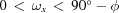, 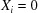 and 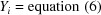, where
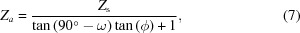


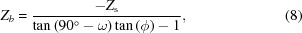


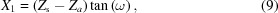


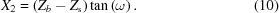



Furthermore, the scattering angle for the *i*th intensity, 2θ_*i*_, found within the *xz* plane can also be determined by
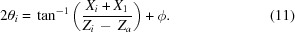



Thus, equation (11) can be used to determine the Bragg angle for any diffraction maxima within the *xz* plane. Corresponding equations can be derived if the specimen rotation is around the *x* axis. Thus, this analytical model shows that parafocusing reflection FCG data can be acquired via a fixed-sample nonlinear detector translation that does not require a fixed relationship between the X-ray source/specimen and specimen/detector.

The proposed incident beam topography can also be applied equally well to both θ:2θ and θ:θ Bragg–Brentano arrangements. The change in minor/major axis footprint ratio required as θ is changed [see equations (1) and (2)] can be approximated by a static angular collimator (see *Results* [Sec sec4]).

This unconventional topology may perhaps be more practically applicable to energy-dispersive X-ray diffraction, where the parafocusing condition afforded by equations (1) and (2) is maintained for multiple scattering angles and fixed X-ray source and detector positions. Furthermore, any peak broadening caused through the use of a circular collimator is significantly less than that arising from the detector energy resolution and finite receiving window of the detector.

To explore this new approach for achieving parafocusing geometry, a combination of experiment and simulation has been employed. Initially, intensity gains from the extended interaction volume are demonstrated, and then specimen/detector rotation and detector translations are compared. Energy-dispersive data acquisitions are subsequently performed to illustrate the circular collimator approximation.

## 3. Material and methods   

### 3.1. Experimental   

A water-cooled sealed X-ray tube (40 kV, 30 mA) with a molybdenum target was used as the X-ray source. The output was filtered (Zr) to produce a quasi-monochromatic beam with energy ∼17.5 keV. A diverging pencil-beam profile was generated with a pinhole collimator (0.5 mm diameter and 1 mm thickness). A hollow conical beam (required for FCG) was produced with an annular collimator (2.11 and 2.62 mm inner and outer radius, respectively, and 1 mm thickness). An area detector (Princeton Instrument PIXIS 1024) was employed to record the spatially resolved diffraction data and an energy-dispersive point detector (X-123CdTe Amptek) used to measure energy-dispersive signatures. The area detector had a 13.3 × 13.3 mm active area containing 1024 × 1024 pixels. All components were mounted on Thor Labs motorized stages to provide translation and rotation movement for each of the system components. A NIST standard reference material (SRM1976), sintered corundum (Al_2_O_3_) and aluminium (Al) were used as the specimens in plate form to illustrate the proof of concept.

The system described above was used to acquire diffractograms using pencil and conical beams, where the detector was rotated at twice the rate of the specimen around a central axis (typical θ:2θ Bragg–Brentano arrangement). In a second series of experiments, the angle between the specimen surface and incident beams was fixed and parafocusing was maintained through a translation of the detector (see Section 2). It should be appreciated that a point detector would have been sufficient to undertake these studies but a more intuitive understanding of the scattering distributions is secured using the area detector.

### 3.2. Simulation   

Simulations were performed using the Monte Carlo ray-tracing hybrid software *McXtrace* (Bergback Knudsen *et al.*, 2013 ▸). A model of the laboratory X-ray tube (above) and its output as a hollow conical beam and pencil beam was produced. All of the collimation elements of the experiments (see above) were reproduced within *McXtrace*, and 0.5 mm-thick plates of Al_2_O_3_ and Al were included to represent the specimens. All of the subsequent diffraction data were captured with detectors having characteristics matching those of the PIXIS 1024 or Amptek X-123CdTe.

## 4. Results and discussion   

### 4.1. Experimental proof of concept   

**Figure 4:**
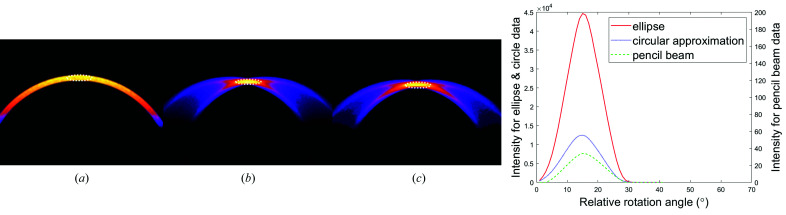
Simulated reflection FCG images illustrating the convergence of scattering maxima from reflection 111 from an aluminium plate as acquired with (*a*) a pencil beam, (*b*) an elliptical collimator and (*c*) a circular collimator. (*d*) Intensity changes are quantified in a relative arbitrary scale. Note: data have been integrated around the white dotted lines as the specimen was rotated by a range of angles ω.

A qualitative comparison between the use of pencil-beam, circular and elliptical collimators was performed by simulation. Fig. 4 ▸ shows the scattering from the 111 reflection of aluminium using a pencil beam, circular (approximation) collimation and elliptical collimation to illuminate the specimen. The experimental geometry was such that the Bragg angle was fixed for this Al reflection. The enhanced confinement of the Bragg intensity for the elliptical collimation is apparent, although the circular collimation appears a good approximation.

**Figure 5:**
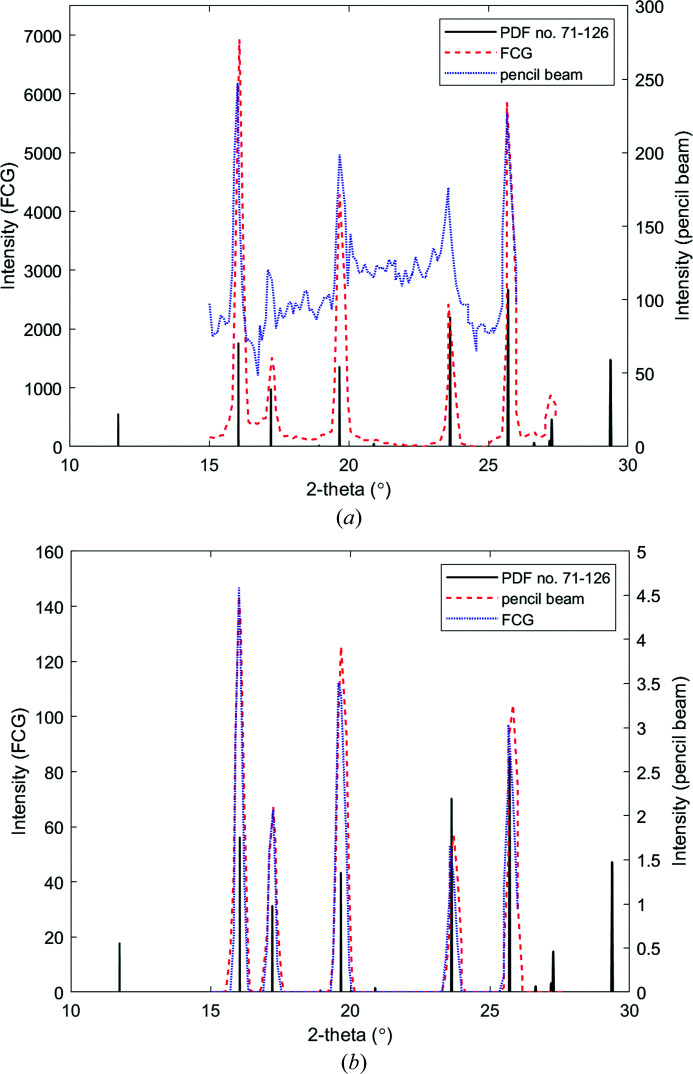
(*a*) Experimental and (*b*) simulated scattering profiles acquired from a 0.5 mm-thick corundum specimen in a θ:2θ reflection mode geometrical arrangement with a pencil beam and a hollow conical incident beam (λ = 0.7107 Å). The standard peaks of corundum are provided for reference (ICDD PDF card No. 71-126).

To determine the intensity gain afforded by the extended interaction volume resulting from annular collimation, experimental and simulated diffraction data were acquired from the NIST sintered aluminium oxide plate using a pencil beam and a circular annular collimator. In each case, the specimen and detector were rotated around a single axis in a θ:2θ ratio. Experimental and simulated results are shown in Figs. 5 ▸(*a*) and 5 ▸(*b*), respectively. As expected because of the increased interaction volume provided by the annular cone beam, the intensity of the Bragg maxima produced from FCG is greater than that produced by the pencil beam by a factor of ∼35 (there is a dependence on 2θ). This finding is in agreement with the theoretical prediction of ∼40 based on differences in footprint areas without considering any intensity correction factors (Prokopiou, 2014 ▸).

**Figure 6:**
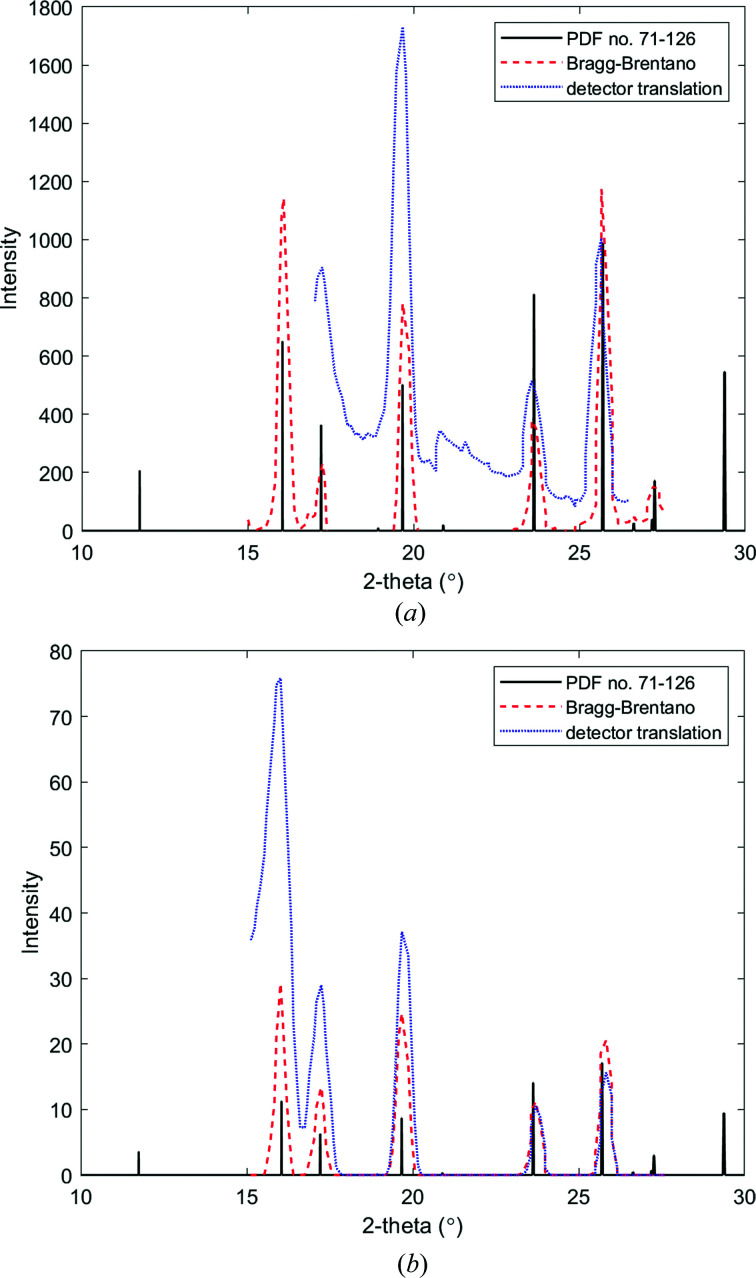
(*a*) Experimental and (*b*) simulated FCG scattering profiles acquired from the NIST corundum plate specimen via a θ:2θ acquisition and a nonlinear detector translation (λ = 0.7107 Å). The standard peaks of corundum are provided for reference (ICDD PDF card No. 71-126).

Experimentally, data collected from a detector translation experiment [using equations (5) and (6)] are illustrated in Fig. 6 ▸(*a*) and the corresponding simulated data in Fig. 6 ▸(*b*). For comparison, data from the θ:2θ experiments are also provided in these plots. A difference in intensity is observed between the two methods at low scattering angles (and an increase in background) as a result of the reducing specimen–detector radial distances within the translation experiments.

### 4.2. Energy-dispersive mode   

**Figure 7:**
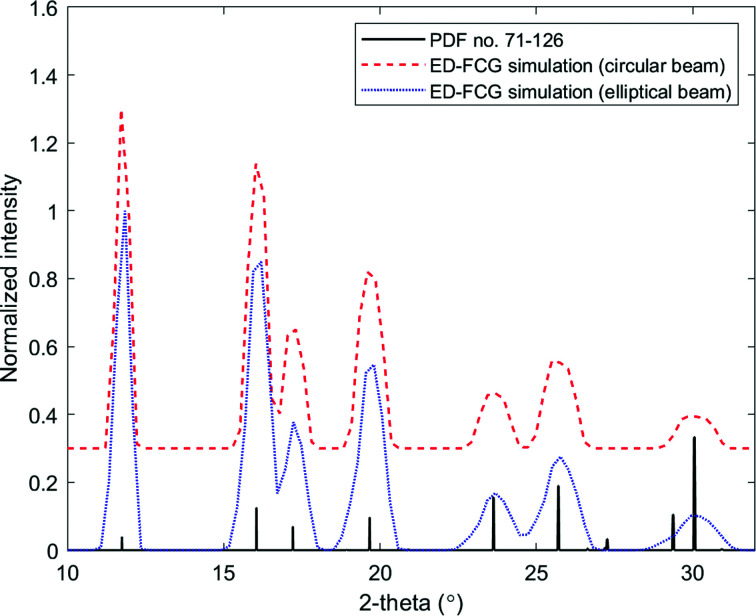
Simulated energy-dispersive FCG scattering profiles acquired from the NIST corundum plate specimen via a θ:2θ parafocusing acquisition with a circular collimator and an elliptical collimator. The standard peaks of corundum are provided for reference (ICDD PDF card No. 71-126). Note that the data have been normalized and the circular-beam diffractogram offset for clarity.

The practical advantages of an energy-dispersive approach were explored through a number of simulations and experimental work. Simulations of parafocusing FCG have been produced in energy-dispersive mode with both circular and elliptical collimation optics and these data are presented in Fig. 7 ▸. Data are presented as a function of 2θ for direct comparison with previous data that were acquired in an angular-dispersive mode. Photon energy has been converted to its angular equivalence by applying equation (12):
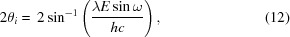
where λ = 0.7107 Å, *E* is the energy in ångström, *h* is Planck’s constant and *c* is the speed of light.

**Figure 8:**
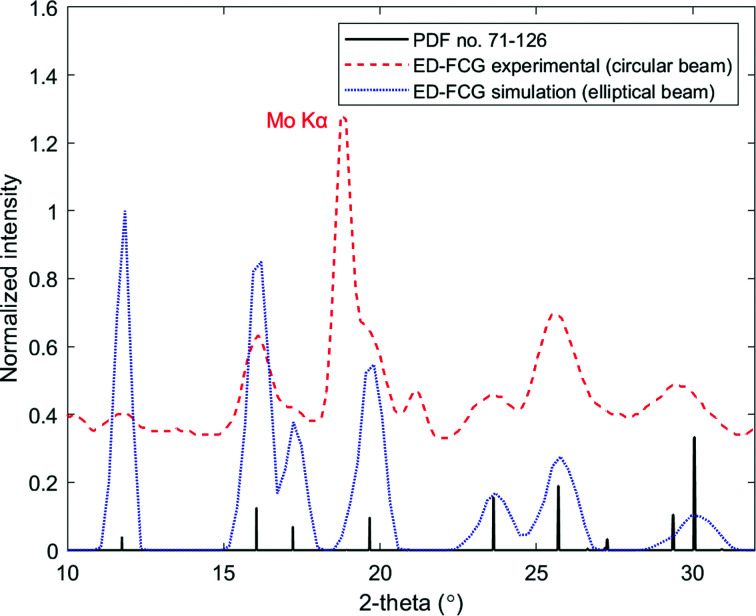
Energy-dispersive FCG scattering profiles acquired from the NIST corundum plate specimen via a θ:2θ parafocusing acquisition with a circular collimator (experimental data) and an elliptical collimator (simulated data). The standard peaks of corundum are provided for reference (ICDD PDF card No. 71-126).

The resulting profiles are, within experimental error, in good agreement with respect to peak position and relative intensities. Owing to practical limitations, experimental energy-dispersive data have been obtained only with a circular collimator. Fig. 8 ▸ shows such a diffractogram from the NIST plate. Note that no background corrections were applied and the additional peak in the diffractogram is the Mo *K*α line from the incident X-ray source.

## 5. Conclusions   

We have been able to demonstrate, for the first time, that parafocusing diffraction geometry can be produced from an extended X-ray beam, with no flat-specimen approximation and data collected by a single-point detector. This method is shown to offer significantly greater diffraction intensities as a result of the increased interaction volumes and, as such, may be applied to reduce data collection times or enhance signal-to-noise ratio. We have replaced the conventional incident beam (pencil or line) with a hollow cone beam where every point on the beam footprint provides a constant scattering angle to a point detector. An advantage of the hollow beam over conventional Bragg–Brentano geometry is that the diffracted intensity can be measured with a significantly smaller detector, as in the conventional case the detector length needs to be similar to that of the beam length on the specimen. Thus, the hollow beam can be extended in radius significantly (to increase intensity) without incurring the detector area penalty.

Laboratory-based data have been acquired and compared with simulations, illustrating the advantages of an annular incident beam in a Bragg–Brentano parafocusing geometry over a conventional pencil beam. The gain in scattered beam intensity is significant when compared with data obtained with a conventional pencil beam under the same experimental conditions. We have also shown that the ideal elliptical collimation can be replaced with a simpler, circular collimator.

An analytical model has also been developed to describe the parafocusing FCG configuration and provide information on the *xyz* coordinates of scattering foci for any given 2θ angle. This approach assisted in developing an alternative method for the acquisition of parafocusing FCG maxima, involving a translation of the detector at a fixed specimen position and rotation angle. This method offers an enhanced intensity of diffraction distributions with low scattering angles as a result of the short specimen–detector radial distances at such angles.

Ultimately, the utility of this method will probably be exploited by adopting an energy-dispersive approach to the data collection, as this achieves the parafocusing from flat specimens without requiring any detector and/or specimen movement. To this end, we have also presented some initial data to support this view.
